# Fall Prevention Knowledge, Attitudes, and Behaviors: A Survey of Emergency Providers

**DOI:** 10.5811/westjem.2020.4.43387

**Published:** 2020-07-10

**Authors:** Kathleen Davenport, Amy Cameron, Margot Samson, Jiraporn Sri-on, Shan W. Liu

**Affiliations:** * University of North Carolina Medical Center, Department of Emergency Medicine, Chapel Hill, North Carolina; † Massachusetts General Hospital, Department of Emergency Medicine, Boston, Massachusetts; ‡ University of Central Florida, College of Medicine, Orlando, Florida; § Navamindradhiraj University, Geriatric Emergency Medicine Research Unit, Bangkok, Thailand

## Abstract

**Introduction:**

Falls are a frequent reason geriatric patients visit the emergency department (ED). To help providers, the Geriatric Emergency Department Guidelines were created to establish a standard of care for geriatric patients in the ED. We conducted a survey of emergency providers to assess 1) their knowledge of fall epidemiology and the geriatric ED guidelines; 2) their current ED practice for geriatric fall patients; and 3) their willingness to conduct fall-prevention interventions.

**Methods:**

We conducted an anonymous survey of emergency providers including attending physicians, residents, and physician assistants at a single, urban, Level 1 trauma, tertiary referral hospital in the northeast United States.

**Results:**

We had a response rate of 75% (102/136). The majority of providers felt that all geriatric patients should undergo screening for fall risk factors (84%, 86/102), and most (76%, 77/102) answered that all geriatric patients screened and at risk for falls should have an intervention performed. While most (80%, 82/102) answered that geriatric falls prevention was very important, providers were not willing to spend much time on screening or interventions. Less than half (44%, 45/102) were willing to spend 2–5 minutes on a fall risk assessment and prevention, while 46% (47/102) were willing to spend less than 2 minutes.

**Conclusion:**

Emergency providers understand the importance of geriatric fall prevention but lack knowledge of which patients to screen and are not willing to spend more than a few minutes on screening for fall interventions. Future studies must take into account provider knowledge and willingness to intervene.

## INTRODUCTION

Falls are common among the geriatric population. Annually, one in every three adults over the age of 65 living in the community falls.^1^ These older adult fallers make up three million visits to the emergency department (ED) each year and represent 10% of the ED visits among that cohort.^2^,^3^ Direct medical care costs due to falls have been estimated to be $200 million for fatal and $19 billion for non-fatal, fall-related injuries.^4^ ED fall patients experience high rates of adverse events.^5^ This places a significant burden on emergency clinicians given the frequency with which the ED cares for such patients.

In an attempt to standardize geriatric ED care, the Geriatric Emergency Department Guidelines were created.^6^ One of the areas of focus was care for geriatric patients following a fall. The guidelines recommend implementing a fall risk assessment tool, using a multidisciplinary team that includes physical therapy, occupational therapy, social work, nursing, and physicians to arrange expedited outpatient follow-up.^6^ However, current ED practice is not concordant with the ED fall guidelines.^7^ It is not clear whether providers do not know about the guidelines or they do know but feel the guidelines are too cumbersome in their already time-constrained practice.^7^

To design a successful ED fall intervention, it is important to determine emergency providers’ level of knowledge and practices and what they are willing to do for fall patients while they are in the ED. We conducted a survey of emergency providers including staff, residents, and physician assistants (PA) to assess their knowledge of fall epidemiology and the geriatric ED guidelines as well as to gather information on current ED practice for geriatric fall patients. Additionally, we asked providers which patients they thought should be evaluated for fall risk and how much time they were willing to dedicate to fall-prevention interventions.

## METHODS

We conducted an anonymous survey in June–August 2017 of emergency providers including attending physicians, residents and PAs at a single, tertiary, Level 1 trauma center located in the northeast United States that sees approximately 100,000 adult patients per year, 25% of whom are over the age of 65. The survey was designed and administered using Redcap (Research Electronic Data Capture) a secure, web-based, electronic data capture tool. Fall experts and qualitative survey experts reviewed and provided feedback on the survey instrument. It was edited accordingly and subsequently piloted among attendings and PAs and ultimately approved by our institutional review board.

We obtained the names of all attendings, residents, and PAs who worked in the ED, using departmental and hospital e-mail listservs. We excluded PAs and attendings who worked per diem or did not see patients clinically. We then emailed each provider with information about the survey and sent two follow-up participation reminders on June 30, 2017, and July 19, 2017. The survey design and sampling methods were conducted in accordance with the guidelines described by Mello et al in a commentary on surveying in emergency medicine (EM).^8^ We compared the difference in proportions of the responses stratified by type of ED providers using chi-square or Fisher’s exact analysis.

## RESULTS

As displayed in Table 1, we had a response rate of 75% (102/136). Of the 102 respondents, 33 (32%) were attending physicians, 38 (37%) were resident physicians, and 31 (30%) were PAs. Non-responders were primarily residents (33%, 20/60) followed by attending physicians (23%, 10/43) and PAs (1%, 3/33). When stratified by type of provider there were no significant differences in responses except for vision (p-value 0.012), orthostatic blood pressure measurement (p-value 0.030), strength/absence or presence of peripheral neuropathy (p-value 0.010), and ensuring a home safety assessment (p-value 0.036).

In terms of knowledge about falls, most respondents overestimated the frequency with which older adults fall (which is approximately a third of the time).^1^ When answering the question “On average, what percentage of community dwelling patients >65 years of age fall annually?” 44% (45/102) answered 34–50%, while 13% (13/102) answered more than 50%. Our survey also showed that the vast majority of respondents were not familiar with the Geriatric ED Guidelines with 66% (67/102) and 32% (33/102) reporting being not at all familiar or only somewhat familiar with them.

Regarding fall screening, interestingly, the overwhelming majority of respondents, 84% (86/102), answered that all patients should undergo screening for fall risk factors, while 15% (15/102) felt only people who come to the ED with a recent fall (two weeks or less) should be screened. Only 1% (1/102) answered that only patients with extremely high risk for future falls should undergo screening. Furthermore, in response to the question “On which geriatric patients should emergency clinicians intervene?” most survey participants (76%, 77/102) answered that all geriatric patients screened and at risk for falls should have an intervention, while 23% (23/102) answered all geriatric patients who present after a fall should have an intervention.

While respondents felt it was important to prevent falls, most were not willing to spend more than five minutes to do so. When asked “How important is it to you to prevent recurrent falls among elderly ED patients?” 80% (82/102) answered very important and 3% (3/102) answered slightly important, indicating that participants at least think that fall prevention is important. Unfortunately, when asked “How much time would you be willing to spend on a fall risk assessment and prevention tool?” only 1% (1/102) reported being willing to spend > 10 minutes, 6% (6/102) were willing to spend 6–10 minutes, and 44% (45/102) were willing to spend 2–5 minutes with the rest reporting only being willing to spend < 2 minutes (46%, 47/102) or no time 3% (3/102).

Our respondents then reported the three major barriers to implementing geriatric falls prevention. The overwhelming response was “Not enough time” (87%, 89/102), followed by “Do not know how to intervene” (51%, 52/102), “No ED resources to intervene” (47%, 48/102), and “Inadequate training on fall evaluation/prevention” (45%, 46/102).

## DISCUSSION

This survey demonstrates that ED providers understand the importance of fall prevention in the older population but lack knowledge of specific screening tools or interventions to prevent future falls in their patients. Our results also demonstrate a lack of knowledge about fall epidemiology and the existence of the Geriatric ED Guidelines, likely explaining why compliance with the guidelines is poor.^7^ While providers occasionally ask some fall-specific questions or conduct some type of intervention, fall interventions are not done on the majority of patients. This likely is due to a lack of consistency in the amount of EM geriatric training in residency and a lack of knowledge of fall guidelines.^9^

While our statistical analysis could only detect an overall difference in frequency of responses across providers on whether they tested vision, orthostatic blood pressure, strength/absence or presence of peripheral neuropathy and performed a home safety evaluation, it appears that when providers respond “all the time” this was largely due to PAs’ responses. This could be due to falls training that PAs may have received in PA school or other geriatric-focused conferences or training. However, McEwan et al concluded that improving education on falls, creating easy access to protocols and guidelines, and having the senior staff mentor junior staff on the screening and interventions led to greatest compliance.^10^ Hence, improving fall training for providers should improve ED fall intervention.

More research into which patient population would most benefit from screening and interventions is needed. Interestingly, most emergency providers in our study thought all patients should be screened and intervened upon but then admitted not knowing whom to screen or how to intervene. While clearly certain patients should be excluded from screening because they are too sick, the question remains which patients must be screened and who would benefit the most from an ED intervention. Another challenge is determining which screening tool to use. A recent meta-analysis of ED-based, fall risk stratification instruments was unable to provide a single best fall screening strategy.^11^ It did find that the ideal fall risk screening instrument would be brief, easy to use by all clinical staff, and would not require additional space or equipment for screening.^11^

A few screening tools have been validated for ED use, but currently there is no agreed-upon tool. One screening tool that can be used is the Centers for Disease Control and Prevention Stopping Elderly Accidents Deaths and Injuries (CDC STEADI), which recommends using three brief screening questions routinely for patients over the age of 65.^12^ The questions are: 1) Have you fallen in the past year? 2) Do you feel unsteady when standing or walking? and 3) Do you worry about falling? If a patient answers “yes” to any of the three questions they are at increased risk of falling and further assessment is recommended.^12^ Follow-up information regarding exercise classes to improve balance and ways to enhance home safety should also be given to the patient. One small, ED-based study by Greenberg et al provided patients in the intervention arm with a CDC STEADI brochure with standardized information about controlling risk factors for falls and found that 12% of patients in the intervention arm started an exercise class and had their medications checked by their primary care provider compared to none in the control arm.^13^ Among intervention patients, 85% (22/26) chose a fall prevention strategy compared to 25% (6/24) in the control group (p<.001). The study did not examine outcome such as repeat falls or ED return visits.

Multifactorial fall-intervention programs have also had mixed outcomes. The landmark PROFET study found that an intensive fall-intervention program significantly reduced fall risk and led to the implementation of many multifactorial fall-prevention programs.^14^ However, follow-up studies results have been mixed. Morello et al did a systematic review and meta-analysis that included 12 randomized control trials of patients aged 60 and older who presented to the ED after a fall.^15^ Included studies had to have a multifactorial falls-prevention intervention and examine at least one falls-related outcome such as recurrent fall, repeat ED visit, or subsequent hospitalization.^15^ Their analysis concluded that there is little evidence that multifactorial falls-prevention interventions reduce falls in older ED patients.^15^

In a different systematic review and meta-analysis by Hopewell et al, 41 randomized control trials of patients 65 and older who lived in the community and presented to the ED after a fall were examined. They concluded that multifactorial fall interventions did reduce falls in the intervention groups, but given the considerable heterogeneity their confidence in the results was low.^16^ These mixed results of fall intervention programs are likely due to the complex physiology of falls, limitations in resources, and difficulty standardizing a process when every healthcare system functions differently. A root cause analysis of why certain programs do not succeed or what factors contributed to another program’s success is needed to provide more guidelines for implementing fall prevention programs in EDs.

While most respondents thought that all geriatric patients should be screened and intervened upon in the ED, this was not how most providers practice. This finding shows a disconnect between what providers think is important and what they are able to accomplish in practice and creates a major challenge in implementing screening and fall interventions. Perhaps most revealing is that most providers are only willing to spend less than five minutes on an intervention. Any successful ED-based intervention needs to be concise or not dependent on the main ED provider. It remains to be seen whether it is practical or efficient to intervene on all geriatric patients, only those who had a fall, or those at highest risk of falling.

## LIMITATIONS

This study was done at one academic ED in an urban setting; therefore, the survey results may not be generalizable. We had a response rate of 75% and therefore may have missed the opinions and input of other providers who did not respond to the survey. The lowest response rates were from resident physicians likely due to their schedule being the most demanding. The fact that most providers felt that all patients should be screened may be due to the social desirability bias. However, this seems less likely as the respondents were frank about the small amount of time they were willing to dedicate to a falls intervention.

## CONCLUSION

Geriatric fall patients are a growing population that will continue to present to the ED. Results of this survey indicate that emergency providers understand the importance of fall prevention in older adults but lack knowledge of which patients to screen and how to prevent future falls. This is likely due to both lack of education and no standard ED fall screening/intervention program. Successful interventions will need to be short, supported by the staff, and not dependent solely on the emergency provider. Research into ED-based fall-prevention screening tools and interventions are needed to help create and implement future guidelines.

## Figures and Tables

**Table 1:** Emergency Department Provider Survey Responses to how frequently they ask about fall risk factors (N=102).

Question/measurement													

When obtaining a history from an older ED fall patients, how often do you do the following?	Never, N(%)				Sometimes, N(%)				All the time, N (%)				
	Total N=102	Attending	Resident	PA	Total N=102	Attending	Resident	PA	Total N=102	Attending	Resident	PA	P-value
Ask about previous falls?	2(2)	0	1(50)	1(50)	72(71)	24(33)	27(38)	21(39)	28(27)	9(32)	10(35)	9(32)	0.964
Ask about difficulty with gait and/or balance?	5(5)	2(40)	3(60)	0	59(58)	20(34)	25(42)	14(24)	38(37)	11(29)	10(26)	17(44)	0.094
Ask about syncope symptoms?	0	n/a	n/a	n/a	46(45)	14(30)	19(41)	13(28)	56(55)	19(34)	19(34)	18(32)	0.745
Ask about specific comorbidities such as dementia, Parkinson’s stroke, diabetes, hip fracture or dementia?	8(8)	2(25)	4(50)	2(25)	70(69)	20(29)	28(40)	22(31)	24(24)	11(46)	6(25)	7(29)	0.505
Ask about patient’s vision?	27(27)	10(37)	12(44)	5(19)	60(59)	23(38)	19(32)	18(30)	15(15)	0	7(47)	8(53)	0.012
Ask about difficulties with activities of daily living?	14(14)	6(43)	6(43)	2(14)	70(69)	21(30)	27(39)	22(31)	18(18)	6(33)	5(28)	7(39)	0.584
Ask about type of footwear used?	70(69)	21(30)	27(39)	22(31)	30(30)	12(40)	9(30)	9(30)	2(2)	0	2(100)	0	0.469
Review patient medications specifically for fall risk?	26(26)	9(35)	11(42)	6(23)	68(67)	22(32)	25(37)	21(31)	8(8)	2(25)	2(25)	4(50)	0.751

**When doing a physical exam on an older ED patient, how often do you do the following?**	Never, N(%)				Sometimes, N(%)				All the time, N (%)				
	Total N=102	Attending	Resident	PA	Total N=102	Attending	Resident	PA	Total N=102	Attending	Resident	PA	P-value

Measure orthostatic blood pressure on fall patients	36(35)	16(44)	15(42)	5(14)	64(63)	17(27)	22(34)	25(39)	2(2)	0	1(50)	1(50)	0.030
Assess strength and presence/absence of peripheral neuropathy	16(16)	8(50)	5(31)	3(19)	64(63)	24(38)	23(36)	17(27)	22(22)	1(5)	10(45)	11(50)	0.010
Evaluate patient gait	0	n/a	n/a	n/a	72(71)	24(33)	31(43)	17(24)	30(29)	9(30)	7(23)	14(47)	0.050
Conduct functional testing (e.g. Get-up-and-go test)	55(54)	18(33)	22(40)	15(27)	43(42)	14(33)	16(37)	13(30)	4(4)	1(25)	0	3(75)	0.424
Consult or refer to Physical Therapy/Occupational therapy for fall patients	0	n/a	n/a	n/a	64(63)	19(30)	29(45)	16(25)	38(37)	14(37)	9(24)	15(39)	0.081
Ensure patients have home safety assessments done	21(21)	3(14)	13(62)	5(24)	67(66)	25(37)	23(34)	19(29)	14(14)	5(36)	2(14)	7(50)	0.036
Recommend exercise	30(30)	12(40)	8(27)	10(33)	64(63)	19(30)	26(40)	19(30)	8(8)	2(25)	4(50)	2(25)	0.666

*ED*, emergency department; *PA*, physician assistant.
